# Translational Pharmaco-Nutritional Approaches in the Management of Clinical Acute Pancreatitis—A Narrative Review

**DOI:** 10.3390/ph18111621

**Published:** 2025-10-27

**Authors:** Muhammad Shamoon, Sara Alzaanin, Safia Naz, Paul N. Smith, Rachel W. Li

**Affiliations:** 1 Division of Metabolism, Endocrinology & Diabetes, Department of Internal Medicine, University of Michigan, Ann Arbor, MI 48109-1079, USA; 2 Division of Immunology and Infectious Diseases, The John Curtin School of Medical Research, The Australian National University, Canberra, ACT 2601, Australia; 3 Trauma and Orthopaedic Research Laboratory, School of Medicine and Psychology, Australian National University, Canberra, ACT 2601, Australia; 4 Department of Pharmacy, Iqra University, Karachi 75500, Pakistan

**Keywords:** acute pancreatitis, therapeutics, pharmacological agents, nutrition, probiotics

## Abstract

Acute pancreatitis (AP) is an inflammatory disorder of the pancreas that can lead to serious systemic complications. Its clinical presentation varies widely, ranging from mild, self-limiting symptoms to severe, life-threatening illness. Currently, there are no specific therapies approved for the treatment of AP, and management primarily relies on supportive care. However, a growing number of clinical trials have evaluated the translational potential of effective therapies derived from experimental models and have identified promising pharmacological agents that may help ameliorate disease severity. Alongside pharmacological approaches, nutritional management of AP has been gaining increasing attention. Evidence supports the use of enteral nutrition over parenteral feeding, as it is associated with a lower risk of necrotic infections, multiple organ dysfunction, mortality, and other associated complications of AP. In this review, we summarize the therapeutic potential of pharmacological and dietary/nutritional interventions, including naturally occurring bioactive compounds, for AP in the context of its molecular pathology, with the aim of supporting improved clinical decision-making, enhancing patient outcomes, and informing future research directions.

## 1. Introduction

Acute pancreatitis (AP) is a common and potentially lethal inflammatory condition of the pancreas [1], a vital glandular organ. Although regional variation exists [2], the global incidence of AP has alarmingly increased over the past few decades, rising by 59% between 1990 and 2021 [3]. Currently, AP affects approximately 35 individuals per 100,000 population annually, equating to nearly 2.75 million people worldwide each year [3]. In the United States alone, AP results in over 255,000 hospitalizations annually, with associated healthcare costs exceeding US $2.5 billion [4]. Moderate to severe AP develops in approximately 20% of patients and carries a mortality rate of 20 to 40% [5,6], further contributing to the economic burden on the healthcare system.

AP presents with varying degrees of severity and is classified accordingly (Table 1) (adapted with slight modifications from reference [7]). The clinical detection of AP is determined by conducting blood tests (e.g., serum amylase, lipase, signs of pancreatic inflammation/infection, C-reactive protein (CRP), etc.) and utilizing the imaging techniques (e.g., ultrasound (US), endoscopic US, computed tomography (CT), magnetic resonance cholangiopancreatography (MRCP)) [8,9]. In most cases, the clinical presentation (i.e., typical radiating abdominal pain) and serum enzymes elevation (at least threefold above the upper limit of normal, with lipase being more specific) are sufficient for the diagnosis, with imaging reserved for uncertain cases or severity assessment. CT-based radiomics is also a pivotal tool in predicting AP and associated organ failure. For instance, recent studies demonstrate that combined radiomics-clinical models achieve enabling earlier risk stratification, personalized management, and more efficient allocation of critical care resources [10,11,12]. Furthermore, early detection and diagnosis of AP remain critical for improving disease outcomes. For instance, timely interventions including rapid fluid resuscitation, adequate pain control, and initiation of early enteral feeding can significantly improve clinical outcomes, reducing morbidity and mortality. Conversely, delays in diagnosis are associated with higher rates of systemic complications and prolonged hospitalization [9,13].

**Figure 1:**
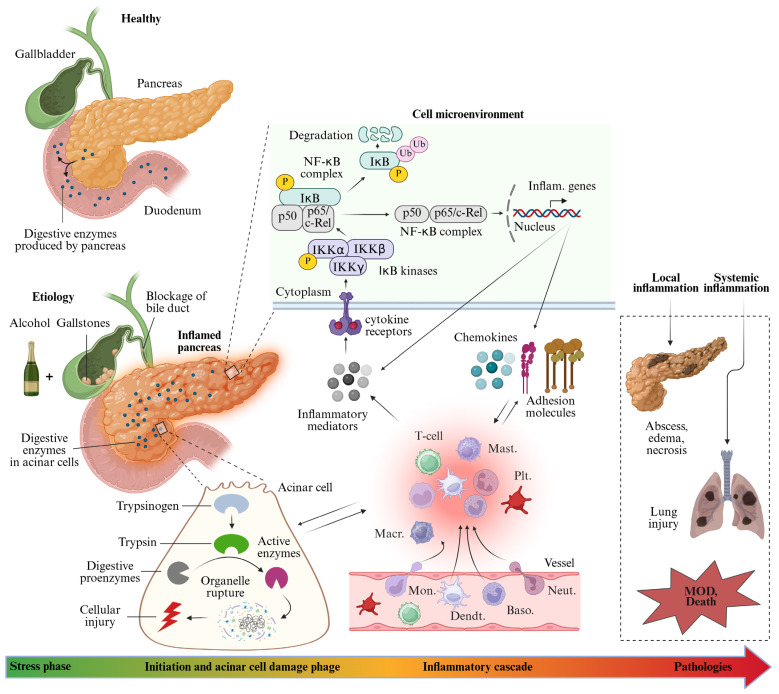
Cellular mechanisms and inflammatory pathways involved in the pathophysiological course of AP. In a healthy pancreas, digestive enzymes are secreted in an inactive form and activated only upon reaching the small intestine. In AP, etiological stress triggers pancreatic injury and premature activation of digestive zymogens. This initiates an inflammatory cascade characterized by immune system activation and the release of various pro-inflammatory mediators. Activated leukocytes further amplify inflammation by increasing the expression of adhesion molecules, promoting leukocyte aggregation and infiltration into pancreatic microcirculation, and releasing additional cytokines and inflammatory mediators. At the cellular signaling level, inflammatory stimuli activate the I kappa B (IκB) kinase complex, which phosphorylates IκBα, leading to its degradation (see introduction for explanation). This process frees nuclear factor-kappa B (NF-κB) to translocate into the nucleus, where it enhances the transcription of inflammatory genes. The resulting upregulation of inflammatory mediators promotes further recruitment of inflammatory cells and sustains the activation of IκB kinase, creating a feedback loop. Meanwhile, platelet-activating factor (PAF) increases the vascular permeability and facilitates the extravasation of inflammatory cells. These combined responses lead to tissue edema, microvascular dysfunction, hypoxia, cellular injury, multiple organ dysfunction (MOD), and ultimately, death. Baso., basophils; Dendt., dendritic cell; IKK, Ikappa kinase; Inflam., inflammatory; Macr., macrophages; Mast., mast cell; Mon., monocytes; Neut., neutrophils; NF-κB, nuclear factor kappa B; P, phosphorylation; Plt., platelet; REL, proto-oncogne; Ub, ubiquitination. Image created with BioRender (www.biorender.com).

The etiology of AP is multifactorial, with gallstones, trauma, metabolic diseases, and alcohol consumption among the most common causes [18]. Before exploring the various treatment interventions for AP, it is essential to develop a comprehensive understanding of its molecular pathology, as outlined here. Cellular events such as pathological Ca^2+^ overload [19], premature activation of digestive enzymes (e.g., trypsin) within acinar cells [20] and macrophages [21], endoplasmic reticulum stress [22], mitochondrial dysfunction [23], impaired autophagy [24], and gut microbiota dysbiosis [25,26] have all been implicated in the pathogenesis of AP. Beyond its pathogenic role, ER stress also represents a potential therapeutic target in AP. Chemical chaperones, PERK pathway inhibitors, and modulators of unfolded protein response signaling have shown promise in preclinical studies, offering opportunities for future AP drug development [27]. These processes contribute to a complex pathophysiological cascade that begins with acinar cell injury, activation of the immune system, and progression to systemic pathological responses (Figure 1). One of the early hallmarks is the premature intra-acinar activation of digestive zymogens, such as trypsin, mediated by enterokinase (Figure 1). This aberrant enzyme activation promotes pancreatic auto-digestion, leading to the release of pro-inflammatory mediators, including tumor necrosis-alpha (TNF-α), interleukin-1 beta (IL-1β), and IL-6, which in turn facilitate crosstalk between acinar and immune cells, further amplifying the inflammatory response [28,29]. The I kappa B (IκB) kinase complex/nuclear factor-kappa B (NF-κB) pathway mediated inflammatory stimuli is a key underlying cellular mechanism of AP [30]. Given its central role in the initiation and propagation of AP, the therapeutic inhibition of IκB/NF-κB pathway has been proposed as a potential target in AP. Numerous experimental models demonstrate that inhibition of IκB/NF-κB axis activity attenuates NF-κB signaling, reduces cytokine release, and alleviates tissue damage [31,32,33].

Collectively, these inflammatory mediators disrupt the pancreatic microcirculation, resulting in increased vascular permeability, edema, hemorrhage and tissue necrosis [34,35] (Figure 1). Consequently, amplified inflammatory reactions, together with extensive acinar cell injury, contribute to the development of life-threatening systemic inflammatory response syndrome (SIRS) [36,37]. SIRS, in turn, leads to distinct organ damage and can progress to multiple organ dysfunction (MOD) [34] (Figure 1), which is ultimately responsible for AP-associated mortality [38].

Although current treatment approaches for AP remain suboptimal, advances in understanding its pathophysiology have driven research toward the development of novel pharmacological and nutritional strategies aimed at restoring organ and tissue homeostasis. Several pharmacological agents [39] have shown promise in targeting key mechanisms of this complex disorder. Alongside pharmacological approaches, the traditional bowl at rest (nothing by mouth) approach has been conventionally employed in the management of AP [7,40]. However, prolonged dietary restriction can exacerbate malnutrition by limiting nutrient intake at a time when the body’s metabolic demands are elevated [41]. This nutritional imbalance may lead to enhanced catabolism, resulting in excessive production of reactive oxygen species and subsequent oxidative stress [42]. These effects can disrupt the gut barrier, promoting bacterial translocation from the gastrointestinal tract to the bloodstream [43], which contributes to infected pancreatic necrosis and increases the risk of mortality [44,45]. Of relevance, pancreatic acinar and Paneth cell-derived antimicrobial peptides (AMPs), including defensins, cathelicidins, lysozyme, and Reg family proteins play a pivotal role in maintaining gut homeostasis and mucosal immunity. These peptides help regulate the microbial composition, prevent bacterial translocation, and sustain intestinal barrier integrity. Whereas dysbiosis of the gut microbiota/AMPs has been linked to immune dysregulation and exacerbation of pancreatic inflammation, including AP, thus underscoring the importance of AMP-mediated gut–pancreas crosstalk in the disease progression [46,47,48]. Strategies aimed at preserving microbiota balance and enhancing endogenous antimicrobial defense; therefore, they may also hold promise in mitigating complications associated with AP.

Thus, both pharmacological and nutritional approaches such as probiotic and antioxidant therapies are equally crucial in the management of AP. Notably, both recent experimental [49,50] and clinical [51] studies suggest that probiotics could help restore disrupted intestinal homeostasis, potentially reducing bacterial translocation and the risk of secondary infection in AP. The present narrative review summarizes the evidence on the therapeutic potential of pharmacological and nutritional strategies in the clinical management of AP in the hopes of supporting better clinical decision-making and patient outcomes.

## 2. Literature Search Methodology

This review was conducted as a narrative review of literature. We performed literature search in PubMed, Scopus, Web of Science, and Google Scholar databases for publications. Only peer-reviewed articles published in English were considered. Search terms, alone or combinations (as appropriate) included: “acute pancreatitis”, “pharmacological therapy/interventions”, “nutritional therapy/interventions” “mild”, “moderate”, “severe”, “critical”, “ERCP”, “NSAIDs”, “antibiotics”, “cytokines”, “immunomodulatory”, “enteral feeding”, “bioactive compounds”, “antioxidants”, “prebiotics” “probiotics”, “clinical trials”, “case reports”, and “translational approaches”.

## 3. Pharmaco-Nutritional Management of Clinical AP

Ongoing research into the pathophysiological course of AP has yielded promising evidence supporting both pharmacological (Table 2) and nutritional interventions (Table 3) to manage disease severity. In the following sections, these therapies have been discussed in detail.

## 4. Pharmacological Approaches

### 4.1. NSAID Therapy in Clinical AP

Non-steroidal anti-inflammatory drugs (NSAID) possess both analgesic and anti-inflammatory effects and are widely used in the treatment of various inflammatory diseases [67], including AP [68]. Most NSAIDs act as non-selective inhibitors of cyclooxygenase (COX) enzymes [67]. Among these, indomethacin and diclofenac are notable agents that can be conveniently administered as rectal suppositories.

In a study involving 117 patients undergoing ERCP, prophylactic administration of indomethacin (100 mg) two hours prior to the procedure significantly reduced the incidence of post-ERCP hyperamylasemia (10.2% vs. 16.2%) and AP (2.5% vs. 6.8%) compared to placebo [69]. This therapeutic benefit of indomethacin was further confirmed in a larger double blind, randomized trial involving 490 patients, where those who received a 100 mg indomethacin suppository immediately before ERCP experienced a significant reduction in the severity of post-ERCP pancreatitis (PEP) [70].

Moreover, recently, in a randomized clinical trial (NCT00820612) of 602 patients, rectal indomethacin treatment significantly reduced the incidence of post-ERCP pancreatitis [71]. Pancreatitis developed in 27 of 295 patients (9.2%) in the indomethacin group vs. in 52 of 307 patients (16.9%) in the placebo group (*p* = 0.005). Similarly, moderate-to-severe pancreatitis developed in 13 patients (4.4%) in the indomethacin group compared with 27 patients (8.8%) in the placebo group (*p* = 0.03).

The potential protective effects of the diclofenac have also been investigated. In a study of 220 patients, rectal administration of diclofenac (100 mg) was associated with a significantly lower frequency of PEP compared to placebo (6% vs. 15%; *p* = 0.05) [72]. Additionally, a prospective randomized control trial involving 104 patients investigated the efficacy of a lower dose of diclofenac (50 mg, reduced to 25 mg in patients over 50 kg body weight [73]. This study reported that PEP pain was significantly reduced in the diclofenac group compared to the control group (7.8% vs. 37.7%; *p* = 0.001), suggesting that even low-dose diclofenac may confer protective benefits.

Furthermore, intramuscular administration of a single 75 mg dose of diclofenac was assessed in a study of 60 patients and was found to significantly reduce the incidence of PEP (*p* = 0.032) [74]. Additionally, they observed a significant increase in the levels of lipoxin A4, resolving D1 and E1 in the diclofenac-treated group compared to the control group *(p* < 0.05), suggesting that this may underlie its protective effect. Recently, this therapeutic benefit of diclofenac was further supported by a larger retrospective study of 301 patients [75]. Although this clinical investigation, which utilized a low dose of 25 mg rectal diclofenac, did not observe a reduction in the incidence of PEP in patients with a native papilla and a body weight under 50 kg, it suggested that a higher dose of rectal NSAIDs, such as 100 mg, should be administered regardless of body weight to prevent PEP [75].

Taken together, the results from these trials support the beneficial role of NSAIDs—particularly indomethacin and diclofenac—in the prevention and attenuation of PEP.

### 4.2. Antibiotics Therapy in Clinical AP

Infected pancreatic necrosis is a major clinical complication that severely worsens prognosis and accounts for approximately 70% of all mortality in AP patients who survive the early phase [76]. The use of antibiotic prophylaxis and therapy in AP has long been debated [77] and current treatment guidelines advocate for minimal and judicious antibiotic usage [78]. A 2004 double-blind, placebo-controlled trial involving 114 patients with AP in combination with a C-reactive protein level exceeding 150 mg/L and/or a CT-verified necrosis, found no significant difference in the incidence of infected pancreatic necrosis between the placebo group and those treated with ciprofloxacin (2 × 400 mg/day) and metronidazole (2 × 500 mg/day) [79].

Nevertheless, antibiotics offer the potential to prevent and/or treat infected necrosis, thereby reducing morbidity and mortality [80,81]. The efficacy of antibiotics depends on their ability to penetrate necrotizing pancreatic tissue, which varies among different antibiotic classes [82], and their activity against the specific bacteria commonly implicated in infected pancreatic necrosis. Given that both imipenem and quinolone demonstrate effective penetration into peripancreatic tissue and offer a broad spectrum of activity against probable pathogens, the selection of antibiotics is typically between them [80,83].

A Cochrane meta-analysis of five randomized controlled trials involving 294 AP patients with CT-verified pancreatic necrosis found that antibiotic prophylaxis significantly reduced mortality (odds ratio 0.37; 95% CI: 0.17–0.83), but not the incidence of infected pancreatic necrosis (odds ratio 0.62; 95% CI: 0.35–1.09) [84]. Sub-group analysis by antibiotic regimen showed that beta-lactams significantly reduced both mortality (odds ratio 0.34; 95% CI: 0.13–0.91) and infected pancreatic necrosis (odds ratio 0.41; 95% CI: 0.20–0.85), whereas quinolone plus imidazole combinations did not.

Similarly, another Cochrane review of seven randomized studies involving 404 AP patients found no significant benefit of prophylactic antibiotics in reducing infection of pancreatic necrosis or mortality [85]. However, imipenem—a beta-lactam antibiotic—significantly reduced the rate of infected pancreatic necrosis (16.8% vs. 24.2%) without significantly affecting mortality. Furthermore, another study randomized 90 patients with acute necrotizing pancreatitis—defined by CT-confirmed necrosis and C-reactive protein levels > 150 mg/L—within 48 h to receive either imipenem (1.0 g plus cilastatin intravenously 3 times a day) or no antibiotic therapy. Early imipenem treatment significantly reduced the need for surgery and the overall incidence of major organ complications (*p* = 0.0003) [86].

Studies on prophylactic use of antibiotics prior to ERCP are limited. Nevertheless, a prospective study by Raty and colleagues [87] involving 321 patients found that administering 2 g of cephtazidime intravenously 30 min before ERCP significantly reduced the incidence of PEP compared to the control group (*p* = 0.009). Based on these findings, prophylactic antibiotics may be considered for routine use prior to ERCP.

Collectively, these findings suggest that while the overall benefit of prophylactic antibiotics in AP remains inconclusive, beta-lactam antibiotics, particularly imipenem, demonstrate superior efficacy in reducing infected pancreatic necrosis, mortality, and major complications. However, to draw more definitive conclusions and determine the most effective antibiotic regimens, future research should prioritize larger, high-quality randomized clinical trials.

### 4.3. Cytokine and Immunomodulatory Therapy in Clinical AP

IL-10 is produced by regulatory immune cells and acts primarily as an anti-inflammatory cytokine [88]. Clinical studies have reported elevated IL-10 levels in patients with both mild and severe AP [89,90]. In a randomized study involving 144 patients, human recombinant IL-10 (4 μg/kg or 20 μg/kg) or placebo was administered 30 min prior to ERCP [91]. IL-10 administration significantly reduced the incidence of PEP compared to placebo (*p* = 0.038). As the study controlled for variables such as age, sex, type of treatment, baseline cytokine levels, the authors concluded that IL-10 independently reduces the risk of PEP.

In addition to IL-10, the immunomodulatory monoclonal anti-TNF-α antibody infliximab, which neutralizes the effects of secreted TNF-α, has been investigated as a potential therapeutic agent in experimental AP [92,93]. In experimental models, blocking TNF-α mediated inflammation with anti-TNF-α antibodies or agents like pentoxifylline has shown beneficial effects on histological score and mortality [92,93,94]. However, clinical data are extremely limited.

To date, only two case reports have described the use of infliximab in patients with AP. A recent case report involving a 48-year-old man with the extremely rare co-occurrence of colitis and AP investigated the therapeutic use of infliximab, administered at 5 mg/kg in three biweekly doses [95]. Treatment led to immediate clinical improvement, including resolution of diarrhea and hematochezia, normalization of pancreatic enzyme levels, and no recurrence of either condition. Similarly, an earlier case described a male patient with segmental Crohn’s disease presenting with severe bloody diarrhea who also developed interstitial AP [96]. Following a single infusion of infliximab, the patient experienced clinical improvement and normalization of serum amylase levels without complications.

These reports highlight the potential of cytokine-targeting therapies, such as infliximab, in the treatment of AP. However, to validate these beneficial effects and determine efficacy and safety in broader patient populations, more well-designed clinical trials are urgently needed. Encouragingly, a randomized trial investigating infliximab for AP (NCT03684278) is currently underway in the UK [97].

Platelet activated factor (PAF) is a phosphoglyceride produced by endothelial cells, macrophages, neutrophils, and platelets [98] and (Figure 1). Alongside pro-inflammatory cytokines (IL-1β, IL-6, IL-8, TNF-α) and anti-inflammatory cytokines (IL-2, IL-10), PAF plays a key role in the pathogenesis of AP [99]. In rodents models, PAF antagonists have been shown to ameliorate the severity of AP [100,101,102].

Numerous clinical studies have also been conducted to evaluate the effect of PAF inhibition in AP. Clinical data suggest that lexipafant (a potent PAF antagonist) could significantly reduce the incidence of pseudocysts, systemic sepsis and deaths when administered within the first 48 h of AP symptom onset [103]. The first clinical trial to assess the efficacy of lexipafant was a randomized, double-blind study involving 83 AP patients who received either placebo or lexipafant at a dose of 60 mg/day intravenously for three days [104]. The inflammatory response over days 1–5 was assessed by measuring IL-8, IL-6, E-selectin, C-reactive protein, and polymorphonuclear elastase-α (1)-antitrypsin. The lexipafant group showed a greater reduction in organ failure (*p* = 0.041), IL-8 (*p* = 0.038), and IL-6 levels. These effects were further confirmed in another clinical trial in which patients received lexipafant (100 mg/day) or placebo for 5–7 days. A significant reduction in organ failure score (OFS) was observed in the treatment group (*p* = 0.003), along with trends toward fewer systemic complications and reduced mortality [105].

However, despite these early promising results, a larger, more definitive multicenter phase III trial failed to demonstrate any benefit of lexipafant in reducing organ failure or mortality in patients with severe AP, suggesting it is unlikely to be effective as a standalone therapy for severe AP [106]. Several factors may explain this discrepancy, including heterogeneity in patient populations, delayed initiation of therapy beyond the early inflammatory phase, and the multifactorial mechanisms underlying AP-associated organ failure. These considerations, again, highlight the challenge of translating promising anti-inflammatory interventions into consistent clinical benefits.

Drotrecogin alfa (Xigris) is a 55 kDa glycoprotein analog of endogenous activated protein C [107]. Low levels of activated protein C are associated with a higher risk of mortality in AP and are thought to influence disease progression by modulating immune and inflammatory responses [108]. Several experimental models of AP treated with activated protein C have demonstrated improved pancreatic histology, decreased infection rates, and lower systemic inflammatory markers [109,110,111].

In clinical settings, drotrecogin alfa has shown potential benefits in the treatment of severe AP and its associated septic complications. The first clinical evaluation in 2004 involved two patients who developed severe sepsis during AP, with treatment resulting in interruption of the severe sepsis cascade and improved organ function [112]. In contrast, a randomized, double-blind study involving 32 patients with severe AP found that intravenous administration of activated protein C (24 µg/kg/h for 96 h) did not result in a significant difference in MOD compared to placebo [113].

Moreover, due to concerns about the potential risk of pancreatic hemorrhage in this population, a prospective safety study was conducted in 166 consecutively admitted patients, of whom 43 met screening criteria and 19 were recruited [114]. In this study, intravenous administration of Drotrecogin alfa (24 µg/kg/h for 24 h) appeared to be safe. Separately, a large randomized, double-blind, placebo-controlled multicenter phase III trial involving 1690 patients with severe sepsis found that Drotrecogin alfa significantly reduced the relative mortality risk by 19.4% (95% CI, 6.6–30.5), and the absolute risk by 6.1% (*p* = 0.005), although it was associated with a trend toward increased bleeding compared to placebo (*p* = 0.06) [115].

These mixed findings underscore the urgent need for larger, well-designed clinical trials to further evaluate the safety, efficacy, and therapeutic potential of immunomodulatory approaches in patients with severe AP. Fortunately, additional immunomodulatory trials are underway, including IL-1 receptor antagonists such as anakinra (NCT04681066) and IL-6 inhibitors such as tocilizumab (NCT06045672), are also undergoing clinical evaluation. Nafamostat mesylate continues to be studied in early AP as a protease inhibitor with anti-inflammatory effects (NCT04419315). Collectively, these ongoing trials highlight the shift toward targeted pharmacological strategies to generate robust evidence from large-scale, multicenter studies before routine implementation.

Autoimmune pancreatitis (AIP) [113], a rare immune-mediated form of pancreatitis is stratified into type 1 (elevated serum IgG4) and type 2 (pancreas specific, and often linked to inflammatory bowel disease). AIP also highlights the therapeutic potential of targeted immunomodulation; however, in contrast to AP, this condition responds rapidly to corticosteroids [116] as a first-line therapy, and to immunosuppression medications (i.e., Azathioprine, Rituximab) at maintenance level [117,118]. These established therapies emphasize that immune-mediated pancreatic inflammation can be specifically targeted, offering a point of contrast with the largely supportive or experimental approaches in AP.

## 5. Nutritional Approaches

### 5.1. Nutrition Therapy in Clinical AP

Historically, AP patients were managed by a nothing by mouth (NBM) strategy to rest the pancreas [119]. Most clinical guidelines recommended withholding oral intake until resolution of abdominal pain, while some also suggested waiting for normalization of pancreatic enzyme levels [13,120,121].

However, early and adequate fluid resuscitation remains the cornerstone of AP management. Lactated Ringer’s solution (LR) is generally preferred over normal saline, as it more effectively corrects metabolic acidosis, attenuates systemic inflammation, and has been associated with reduced risk of organ failure [122]. Further experimental evidence suggests that lactate may signal through G-protein-coupled receptor 81 (GPR81), modulating immune and inflammatory responses during AP [123,124]. While fluid therapy is essential, over-resuscitation carries risks, including abdominal compartment syndrome, pulmonary edema, and worsened outcomes. Therefore, individualized, goal-directed fluid replacement is recommended.

Indeed, in AP, intestinal barrier dysfunction—combined with bacterial overgrowth due to impaired gut motility and systemic immunosuppression—promotes bacterial translocation, leading to pancreatic tissue necrosis and infection, and the development of MODS [43,44,125]. Maintaining gut barrier integrity is a central therapeutic goal in the management of AP [25,126,127]. For this reason, in AP, nutritional support has been proposed to help prevent morphological deterioration of the intestinal lining and restore gut function [7,128].

The metabolic response in AP [129,130] closely resembles that seen in severe sepsis or trauma [131], characterized by increased protein catabolism, persistent gluconeogenesis despite exogenous glucose administration, elevated energy expenditure, insulin resistance, and increased dependence on fatty acid oxidation for energy. These metabolic alterations, combined with the dynamic clinical course of AP, mean that energy and nutrient requirements vary depending on disease severity, stage, patient comorbidities, and complications [132]. The dietary modification for patients with AP typically emphasizes avoidance of alcohol, high-fat foods, and refined sugars, all of which may exacerbate pancreatic inflammation and delay recovery. Instead, a gradual introduction of easily digestible, low-fat diet with adequate protein and complex carbohydrates is recommended, with small frequent meals favored over large portions [40,133,134]. Such a balanced diet approach reduces pancreatic stimulation, supporting nutritional recovery.

The discussion above, thus, suggests nutritional support in AP is essential. Table 3 summarizes the various forms of nutritional therapy and their protective roles across the clinical spectrum of AP, as well as the effectiveness of the two main forms of nutrient delivery: enteral nutrition, which involves the delivery of nutrients directly to the gastrointestinal tract, and parenteral nutrition, which provides nutrients intravenously, bypassing the gastrointestinal tract. Total enteral nutrition is able to attenuate the acute-phase response—as evidenced by reductions in serum C-reactive protein, IgM anti-endotoxin antibodies, and improvements in total antioxidant capacity—and to improve clinical outcomes by mitigating disease severity [135]. It is of relevance that studies have also explored on-demand feeding within 72 h, in which patients begin oral intake as soon as they report hunger. While this approach leverages hunger as a marker of gastrointestinal recovery and offers favorable outcomes to some extent, the superiority of “on demand” oral intake over the enteral nutrition is not established [52].

Enteral feeding could also maintain the gut mucosal barrier and hinder bacterial translocation, thereby limiting the risk of infection in pancreatic necrosis [136,137]. Multiple studies (as summarized in Table 3) have demonstrated that early oral feeding during the course of AP is associated with shorter hospital stays, decreased infectious complications, and lower morbidity and mortality. In contrast, while a small percentage of AP patients will still need a parenteral nutrition, total parenteral nutrition is not recommended for patients with either mild or severe AP [137], as numerous randomized controlled trials have linked total parenteral nutrition to increased risks of infection and other closely related complications.

Beyond conventional nutritional interventions, several studies have explored the use of immune-enhanced naturally occurring bioactives as emerging adjuncts therapy for the management of AP. While these compounds hold promise, most supporting evidence remains preclinical, and rigorous clinical trials are needed to define their role in routine clinical therapy. Curcumin (i.e., from turmeric) and resveratrol (i.e., from grapes and berries) have demonstrated modulation of several key AP signaling pathways and attenuation of pro-inflammatory cytokines release/pancreatic inflammation in preclinical models [138,139,140,141]. Of relevance, results from a recent placebo controlled randomized trial (NCT04989166) [142] also demonstrate a reduction in the gastrointestinal ward length of stay, lower analgesics requirements, and an improved appetite in the patients with mild/moderate AP receiving twice 40 mg soft gel nano-curcumin for 2 weeks.

Quercetin (i.e., from onions and apples) demonstrates protective effects against oxidative stress and acinar cell apoptosis; such that a recent work indicates that quercetin alleviates experimental AP by modulating glycolysis and mitochondrial function via PFKFB3 inhibition [143]. Relevantly, for the treatment of AP, systematic reviews [144] and a meta-analysis [145] also emphasize antioxidant and immunomodulatory potential of quercetin despite bioavailability challenges.

Additionally, omega-3 fatty acids (i.e., from fish oil) modulate immune responses, reduce systemic inflammation, and may improve clinical outcomes in small-scale clinical studies [146,147], thereby reducing the risk of mortality, infectious complications, and length of hospital stay and suggesting a promising adjunctive role in comprehensive nutritional strategies for AP.

### 5.2. Antioxidant Therapy in Clinical AP

Oxidative stress plays a significant pathological role in AP, closely linked to the systemic inflammatory response [148]. Hypo-oxygenated pancreatic tissues and polymorphonuclear leukocytes generate ROS, which can further infiltrate and damage the inflamed pancreas [149]. Clinical studies have demonstrated that blood levels of antioxidants are depleted during AP, with lower levels correlating with increased disease severity [150,151].

Antioxidants such as n-acetylcysteine (NAC), methionine, beta-carotene, selenium, ascorbic acid, and α-tocopherol form a heterogeneous group of agents that modulate the inflammatory response and may help mitigate oxidative tissue damage in inflammatory diseases [152,153,154]. Clinical trials assessing these agents support the role of ROS in pancreatic cellular injury and highlight the therapeutic potential of antioxidant supplementation in AP. One randomized clinical trial evaluated the combined protective effects of NAC (200 mg every 8 h), vitamin C (500 mg every 8 h), and antoxyl forte (1 capsule every hour) in AP [155]. The intervention led to a significant reduction in oxidative stress markers (thiobarbituric acid reactive substances and superoxide dismutase), alongside a marked increase in serum antioxidant levels and total antioxidant capacity. The authors further hypothesized that antioxidant supplementation may decrease the hospital stay duration and complication rates in AP patients.

Also, in another randomized trial (treatment group, N = 19; control group, N = 20), Bansal and colleagues (2011) [156] investigated the effects of intravenous antioxidant therapy—including Vitamins A (10,000 IU; i.m.), C (1000 mg; i.v.), and E (200 mg; oral)—in patients with severe AP. While the therapy was safe and well tolerated, it did not result in significant improvements in clinical outcomes such as systemic inflammatory response, organ dysfunction, hospital stay, or mortality. These findings underscore a potential role for Vitamin therapy (i.e., A, C, and E) in the pathophysiology of AP, but therapeutic supplementation has yet to demonstrate the clear clinical benefits. Therefore, further research, including large-scale trials, is warranted to explore the combined antioxidant therapy, particularly in early stages of AP.

Furthermore, glutamine, a potent antioxidant, is an important constituent of both intra- and extracellular amino acid pools and plays an essential role in the development and function of immune cells [157,158]. Its depletion has been demonstrated in critically ill patients [159]. A meta-analysis of 12 randomized controlled trials of glutamine supplementation in AP showed a mortality benefit and a significant reduction in infectious complications, although no significant difference was observed in length of hospital stay [160]. These findings are supported by another meta-analysis conducted by Jeurnink and colleagues [161], which concluded that glutamine treatment may offer potential benefits for AP patients. Furthermore, early administration (initiated on the day of admission) of alanyl-glutamine dipeptide in cases of severe AP has been associated with statistically significant improvements in key clinical outcomes, including duration of hospitalization, rate of infection, organ dysfunction, need for surgery and mortality, when compared to delayed treatment initiated five days after admission [162].

In summary, these studies indicate that glutamine may represent a promising adjunctive therapy in the management of AP.

### 5.3. Probiotic Therapy in Clinical AP

Changes in intestinal motility, microbiome composition [43], immune response [28], and mucosal barrier function [125] contribute to bacterial translocation [163]—primarily involving Gram-negative strains—which can lead to pancreatic necrosis infection. The exact pathomechanisms and specific routes of this translocation, though remain incompletely understood, are actively being investigated [164,165]. Definitive evidence is also lacking regarding whether bacteria predominantly originate from the colon or the small bowel. Nevertheless, the recognized role of bacterial translocation in the progression of AP has prompted several studies to explore the therapeutic potential of probiotics in reducing necrotic infection. Oral probiotics are living microorganisms that confer health benefits beyond basic nutrition by restoring gut integrity, modulating immune responses to invading pathogens, and inhibiting the proliferation of harmful bacteria [166,167].

A recent review of both experimental and clinical studies suggests that probiotics and/or probiotic food may plausibly diminish bacterial translocation and thereby decrease the risk of infectious complications in AP [168]. These findings have been further supported by clinical evidence. For example, a prospective randomized trial involving 66 patients with severe AP compared standard EN (N = 32) to EN combined with *Bifidobacterium* quadruplex live bacterial tablets (N = 34) [169]. Probiotic supplementation was associated with significant reductions in inflammatory markers such as IL-6, TNF-α, and C-reactive protein (*p* < 0.05 for all inflammatory markers), as well as clinical improvements including relief of abdominal pain, alleviation of pancreatic edema, and shorter hospital stays (*p* < 0.05 for all outcomes) [169].

In another placebo-controlled, double-blind clinical study of 64 AP patients, a combination of *Bacillus subtilis* and *Enterococcus faecium* was evaluated [51]. Although no difference in recurrent abdominal pain was observed between the probiotic and control groups, the probiotic-treated group showed a statistically significant reduction in the time to abdominal relief (*p* < 0.01), time to successful oral feeding (*p* < 0.01), and length of hospital stay (5.36 ± 0.15 vs. 6.02 ± 0.17 d, *p* < 0.05). A prior randomized clinical trial involving 22 patients with AP demonstrated that *Leuconostoc plantarum* 299, administered at a dose of 1 × 10^9^ organisms twice daily for one week alongside oat fiber, significantly reduced pancreatic sepsis and the number of surgical interventions related to pancreatic damage [170].

Additionally, a placebo-controlled double-blind study of 62 patients with severe AP also evaluated a combination of four probiotic strains (*L. mesenteroides*, *L. plantarum*, *L. paracasei*, *Pediococcus pentosaceus*) at a dose of 1 × 10^10^ colony forming units administered once daily for one week along with prebiotics containing four bioactive fibers (inulin, beta-glucan, resistant starch and pectin) [171]. This intervention resulted in a statistically significant reduction in SIRS and multiple organ failure (MOF) compared to the control group receiving only prebiotic feeding (*p* < 0.05), suggesting a protective role of probiotics against organ dysfunction in severe AP.

Moreover, Ecologic 641, a multispecies probiotic preparation containing *L. casei*, *L. salivarius*, *L. acidophilus*, *L. lactis*, *B. bifidum*, and *B. lactis*, was shown to significantly increase levels of the anti-inflammatory cytokine IL-10 and decrease levels of the pro-inflammatory cytokine IL-2, compared to its individual components [172]. These findings further suggest that probiotics may help modulate inflammation in AP. Furthermore, the administration of synbiotics (*L. mesenteroides*, *L. plantarum*, *L. paracasei*, *P. pentosaceus* at a dose of 1 × 10^10^ organisms combined with dietary fibers) in 90 patients with severe AP significantly reduced the rate of pancreatic necrosis infection, the need for surgical interventions, and the length of hospital stay, suggesting that early, low-volume enteral oral synbiotic supplementation could potentially be incorporated into routine treatment protocols for AP [173].

Though promising, existing clinical data are often incomplete such that numerous limitations and questions remain. For instance, the timing of intervention, optimal probiotic strain(s) and/or the clinical practice guidelines for the components of the probiotic foods. We, therefore, encourage the field to move toward using future large, multicenter randomized controlled trials to uncover the mechanisms and efficacy of probiotics to cure the AP. This would help bridge the gap in our understating and enhance the knowledge of potential probiotic interventions in treatment of AP.

## 6. Conclusions

The global incidence of AP continues to rise, posing a significant healthcare burden. Recent advances in understanding the cellular and molecular mechanisms of AP in animal models have led to the identification of promising pharmacological agents, some of which have shown beneficial effects in clinical settings. These pharmacological interventions aim to prevent or treat pancreatic necrosis, multiple organ dysfunction syndrome, and infection of necrotic pancreatic tissue. In parallel, nutritional support has emerged as a key component in the management of AP. Importantly, the first 24–48 h after symptom onset represent a critical interventional window during which inflammatory processes can be effectively targeted, potentially improving clinical outcomes. To advance the field, we propose a conceptual framework that integrates pharmaco-nutritional strategies into a tiered clinical algorithm: (i) early baseline care; (ii) adjunctive therapies tailored by risk stratification (e.g., antioxidants, immunonutrition in severe disease); and (iii) emerging targeted interventions (e.g., cytokine inhibitors, bioactives) within controlled trials. Looking ahead, future progress requires multicenter registries and international collaborative studies to refine patient phenotyping, validate biomarkers of response, and translate preclinical discoveries into practice. This approach will not only consolidate current knowledge but also provide a roadmap for personalized management of AP. Finally, such a globally coordinated effort would offer a robust platform for evaluating emerging therapies and improving care for this complex and often life-threatening condition.

## Figures and Tables

**Table 1:** Classification of acute pancreatitis.

AP Classification	Degree of Severity	Complications			
		Local	Systemic		
			TOF	POF	EPC
Atlanta 1992 [14]	Mild	×	×	×	N/A
	Severe	√	√	√	N/A
* Revised Atlanta 2012 [15,16]	Mild	×	×	×	×
	Moderate	√	√	×	√
	Severe	√	×	√	√ or ×
^#^ Determinant-based [17]	Mild	×	×	×	N/A
	Moderate	Sterile	√	×	N/A
	Severe	Infected	√	√	N/A
	Critical	Infected	×	√	N/A

TOF, transient organ failure; POF, persistent organ failure; EPC, exacerbation of pre-exiting comorbidity; √, Yes; ×, No; N/A, not applicable; * According to the revised Atlanta classification (2012) [15,16], local complications are subcategorized into interstitial oedematous, necrotizing pancreatitis, infected necrotizing pancreatitis, and other local complications. Systemic complications are defined as TOF, POF, or an EPC. POF is defined as a Marshall score of 2 or more in any one of the three organ systems—renal, respiratory, cardiovascular—persisting for more than 48 h. ^#^ The sepsis-related organ failure assessment (SOFA) scoring system is also used to define organ failure. For a diagnosis of severe pancreatitis, either POF or infected pancreatic necrosis is required.

**Table 2:** Summary outline of pharmacological therapies for management of clinical AP.

Therapy	Examples	Evidence/Clinical Implications
NSAIDs	Indomethacin, Diclofenac	Effective prophylaxis in high-risk patients, standard of care in post- endoscopic retrograde cholangiopancreatography (ERCP) settings
Antibiotics	Carbapenems, Quinolones	Guidelines discourage routine prophylaxis
Cytokines	Anti-TNF-α, infliximab	Experimental/under clinical investigation, not in routine practice, inconclusive survival benefits
Immunomodulatory	Platelet activated factor, Lexipafant	Limited therapeutic role

**Table 3:** Nutritional therapeutic management of clinical AP.

Nutrition	Study Design	N	Protective Role(s) in Clinical AP	Conclusion
EEN vs. ODN [52]	RCT	208	■ infection (25% vs. 26%), ↓ death (11% vs. 7%)	EEN showed no significant advantage over ODN in ↓ infection and mortality rates
EEN vs. DEN [53]	PCT, RCT	60	■ IAP, ↓ IAH, beneficial for patients with an IAP < 15 mmHg, ■ mortality	EEN prevents IAH and ↓ the severity of severe AP compared with DEN
SD vs. CLD [54]	RIT	60	(*) ↓ hospitalization stay, (*) ↓ post-refeeding length of hospitalization	A SD as the initial meal in patients with mild AP is well tolerated and ↓ length of hospitalization
EEN vs. DEN [55]	HCS	197	↓ pancreatic necrosis (4 vs. 18), ↓ respiratory failure and transfer to intensive care unit occurred (5 vs. 15), ↓ (9 vs. 16), ↓ surgery (7 vs. 11), (*) ↓ mortality (0 vs. 9)	EEN started within 48 h of admission improves clinical outcomes via reducing complications
TEN vs. TPN [56]	RCT	107	(*) ↓ MOF (21% vs. 80%), (*) ↓ surgery (22% vs. 80%), (*) ↓ pancreatic septic necrosis (23% vs. 72%), (*) ↓ mortality (11% vs. 43%).	TEN is better than TPN in preventing pancreatic necrotic infection
EN vs. TPN [57]	PCT, RCT	50	(*) ↓ serum CRP, (*) ↑ serum albumin, (*) ↑transferrin value, ■ surgery (56% vs. 60%), ■ infective complications (64% vs. 60%), ■ hospital stay, ■ mortality (20% vs. 16%)	EN is comparable to PNT in terms of hospital stay, need for surgical intervention, infections and mortality
EIN vs. TPN [58]	HCS	76	↓ severity, ↑ intestinal permeability, ↑ clinical outcomes	Improved clinical outcomes with EIN compared to TPN
TEN vs. TPN [59]	PRT	22	■ APACHE II score, CRP, TNF-a, IL-6, pre-albumin and albumin levels, ↓ severe complications, ■ surgery, ■ hospital stay	TEN tends to be associated with a better outcome compared to TPN
TEN vs. TPN [60]	RCT	466	(*) ↓pancreatic infectious complications (7 vs. 16), ↓ MOF (7 vs. 17), (*) ↓overall mortality 2 vs. 12)	Early TEN could be used as prophylactic therapy for infected pancreatic necrosis
TEN + Abx vs. TPN + Abx [61]	PNR	87	↓ MOF (31% vs. 79%), ↓ surgery (25% vs. 88%), ↓ pancreatic necrosis infection (20% vs. 74%), (*) ↓ death rate (5% vs. 35%)	TEN could be used as a prophylactic therapy for infected pancreatic necrosis
EN vs. PN [62]	RCT	728	↓ CRP, ■ cholecystokinin levels, ↓ mortality, ↓ infected pancreatic necrosis, ↓ cost	EN tends to be associated with fewer septic complications, quicker inflammation reduction, and greater cost-effectiveness compared to PN
EN *+* PN vs. TPN [63]	RCT	96	↑ body weight and pre-albumin, ↓ APACHE II, ↓TNF-a, ↓ IL-6, ↓ serum CRP, ■ albumin, ■ pancreatic lesions, ■ endotoxin and lactulose/manicol of urine, (*) ↑ CD4:CD8 T-cells and IgG	Combined therapy of EN and PN may be better than TPN as it improves nutrition status, moderates inflammation, and protects the gut integrity and immunity more effectively
TEN vs. TPN [64]	RCT	17	↓ fatigue, ■ oxidative stress, ■ plasma glutamine, ↓ respiratory failure, ↓ hospital stay, ↓ cost	TEN is as safe and as efficacious as TPN
TEN vs. TPN [65]	RCT	156	↓ feeding duration, ↓nutrition costs, (*) ↓ nutritional requirements, (*) ↓ metabolic and septic complications	TEN seems to be safer and less expensive than TPN
TEN vs. TPN [66]	RCT	89	(*) ↓ septic complications, ↓ MOF, ↓ mortality	EEN in combination with abx prophylaxis may prevent MOF

■, no effect; * results are significant; ↑, increase/higher; ↓, decrease/lower; TEN, total enteral nutrition; TPN, total parenteral nutrition; EEN, early enteral nutrition; DEN, delayed enteral nutrition; EIN, eco immune nutrition; SD, solid diet; CLD, clear liquid diet; ODN, on demand nutrition; Abx, antibiotics; vs., comparison; RCT, randomized control trial; PCT, pilot/prospective clinical trial; HCS, hospital conducted study; PRT, prospective randomized trial; PNR, prospective non-randomized; RIT, randomized interventional trial; MOF, multiple organ failure; IAP, intra-abdominal pressure; IAH, intra-abdominal hypertension; CRP, C-reactive protein; APACHE II, Acute physiology and chronic health evaluation II.

## Data Availability

No new data were created or analyzed in this study.

## Abbreviations

The following abbreviations are used in this manuscript:

AP	Acute pancreatitis
TOF	transient organ failure
POF	persistent organ failure
EPC	exacerbation of pre-exiting comorbidity
N/A	not applicable
SOFA	sepsis-related organ failure assessment
TNF-α	tumor necrosis-alpha
IL	interleukin
SIRS	systemic inflammatory response syndrome
MOD	multiple organ dysfunction
Iκ	I kappa
NF-κB	nuclear factor kappa B
PAF	platelet-activating factor
Neut.	neutrophils
Baso.	basophils
Dendt.	dendritic cell
Mon.	monocytes
Macr.	macrophages
Plt.	platelet
Mast.	mast cell
REL	proto-oncogne
P	phosphorylation
Ub	ubiquitination
NSAID	non-steroidal anti-inflammatory drugs
ERCP	endoscopic retrograde cholangiopancreatography (ERCP)
PEP	post-ERCP pancreatitis
NBP	nothing by mouth
TEN	total enteral nutrition
TPN	total parenteral nutrition
EEN	early enteral nutrition
DEN	delayed enteral nutrition
EIN	eco immune nutrition
SD	solid diet
CLD	clear liquid diet
ODN	on demand nutrition
FA	Fatty acids
Abx	antibiotics
Ctl	control
vs.	comparison
RCT	randomized control trial
PCT	pilot/prospective clinical trial
HCS	hospital conducted study
PRT	prospective randomized trial
PNR	prospective non-randomized
RIT	randomized interventional trial
MOF	multiple organ failure
IAP	intra-abdominal pressure
IAH	intra-abdominal hypertension
CRP	C-reactive protein
APACHE II	Acute physiology and chronic health evaluation II
sTNFRI	soluble tumor necrosis factor receptor I.
NAC	n-acetylcysteine
ROS	reactive oxygen species

## References

[1] Lee P.J., Papachristou G.I. (2019). New Insightss into Acute Pancreatitis. Nat. Rev. Gastroenterol. Hepatol..

[2] Iannuzzi J.P., King J.A., Leong J.H., Quan J., Windsor J.W., Tanyingoh D., Coward S., Forbes N., Heitman S.J., Shaheen A.-A. (2022). Global Incidence of Acute Pancreatitis Is Increasing Over Time: A Systematic Review and Meta-Analysis. Gastroenterology.

[3] Li T., Qin C., Zhao B., Li Z., Zhao Y., Lin C., Wang W. (2024). Global and Regional Burden of Pancreatitis: Epidemiological Trends, Risk Factors, and Projections to 2050 from the Global Burden of Disease Study 2021. BMC Gastroenterol..

[4] Peery A.F., Murphy C.C., Anderson C., Jensen E.T., Deutsch-Link S., Egberg M.D., Lund J.L., Subramaniam D., Dellon E.S., Sperber A.D. (2025). Burden and Cost of Gastrointestinal, Liver, and Pancreatic Diseases in the United States: Update 2024. Gastroenterology.

[5] Schepers N.J., Bakker O.J., Besselink M.G., Ahmed Ali U., Bollen T.L., Gooszen H.G., van Santvoort H.C., Bruno M.J. (2019). Dutch Pancreatitis Study Group Impact of Characteristics of Organ Failure and Infected Necrosis on Mortality in Necrotising Pancreatitis. Gut.

[6] Bang J.Y., Wilcox C.M., Arnoletti J.P., Varadarajulu S. (2020). Superiority of Endoscopic Interventions over Minimally Invasive Surgery for Infected Necrotizing Pancreatitis: Meta-analysis of Randomized Trials. Dig. Endosc..

[7] Pan L.-L., Li J., Shamoon M., Bhatia M., Sun J. (2017). Recent Advances on Nutrition in Treatment of Acute Pancreatitis. Front. Immunol..

[8] Sandrasegaran K., Heller M.T., Panda A., Shetty A., Menias C.O. (2020). MRI in Acute Pancreatitis. Abdom. Radiol..

[9] Trikudanathan G., Yazici C., Phillips A.E., Forsmark C.E. (2024). Diagnosis and Management of Acute Pancreatitis. Gastroenterology.

[10] Liu N., Wan Y., Tong Y., He J., Xu S., Hu X., Luo C., Xu L., Guo F., Shen B. (2023). A Clinic-Radiomics Model for Predicting the Incidence of Persistent Organ Failure in Patients with Acute Necrotizing Pancreatitis. Gastroenterol. Res. Pract..

[11] Xue M., Lin S., Xie D., Wang H., Gao Q., Zou L., Xiao X., Jia Y. (2023). The Value of CT-Based Radiomics in Predicting the Prognosis of Acute Pancreatitis. Front. Med..

[12] Qi M., Lu C., Dai R., Zhang J., Hu H., Shan X. (2024). Prediction of Acute Pancreatitis Severity Based on Early CT Radiomics. BMC Med. Imaging..

[13] Tenner S., Vege S.S., Sheth S.G., Sauer B., Yang A., Conwell D.L., Yadlapati R.H., Gardner T.B. (2024). American College of Gastroenterology Guidelines: Management of Acute Pancreatitis. Am. J. Gastroenterol..

[14] Bradley E.L. (1993). A Clinically Based Classification System for Acute Pancreatitis. Summary of the International Symposium on Acute Pancreatitis, Atlanta, Ga, September 11 through 13, 1992. Arch. Surg..

[15] Colvin S.D., Smith E.N., Morgan D.E., Porter K.K. (2020). Acute Pancreatitis: An Update on the Revised Atlanta Classification. Abdom. Radiol..

[16] Banks P.A., Bollen T.L., Dervenis C., Gooszen H.G., Johnson C.D., Sarr M.G., Tsiotos G.G., Vege S.S., Acute Pancreatitis Classification Working Group (2013). Classification of Acute Pancreatitis—2012: Revision of the Atlanta Classification and Definitions by International Consensus. Gut.

[17] Dellinger E.P., Forsmark C.E., Layer P., Lévy P., Maraví-Poma E., Petrov M.S., Shimosegawa T., Siriwardena A.K., Uomo G., Whitcomb D.C. (2012). Determinant-Based Classification of Acute Pancreatitis Severity: An International Multidisciplinary Consultation. Ann. Surg..

[18] Weiss F.U., Laemmerhirt F., Lerch M.M. (2019). Etiology and Risk Factors of Acute and Chronic Pancreatitis. Visc. Med..

[19] Du W., Liu G., Shi N., Tang D., Ferdek P.E., Jakubowska M.A., Liu S., Zhu X., Zhang J., Yao L. (2022). A microRNA Checkpoint for Ca2+ Signaling and Overload in Acute Pancreatitis. Mol. Ther..

[20] Modenbach J.M., Möller C., Asgarbeik S., Geist N., Rimkus N., Dörr M., Wolfgramm H., Steil L., Susemihl A., Graf L. (2025). Biochemical Analyses of Cystatin-C Dimers and Cathepsin-B Reveals a Trypsin-Driven Feedback Mechanism in Acute Pancreatitis. Nat. Commun..

[21] Sendler M., Weiss F.-U., Golchert J., Homuth G., Van Den Brandt C., Mahajan U.M., Partecke L.-I., Döring P., Gukovsky I., Gukovskaya A.S. (2018). Cathepsin B-Mediated Activation of Trypsinogen in Endocytosing Macrophages Increases Severity of Pancreatitis in Mice. Gastroenterology.

[22] Yan C., Ma Y., Li H., Cui J., Guo X., Wang G., Ji L. (2023). Endoplasmic Reticulum Stress Promotes Caspase-1-Dependent Acinar Cell Pyroptosis through the PERK Pathway to Aggravate Acute Pancreatitis. Int. Immunopharmacol..

[23] Liu W., Ren Y., Wang T., Wang M., Xu Y., Zhang J., Bi J., Wu Z., Zhang Y., Wu R. (2024). Blocking CIRP Protects against Acute Pancreatitis by Improving Mitochondrial Function and Suppressing Pyroptosis in Acinar Cells. Cell Death Discov..

[24] Ji L., Wang Z., Zhang Y., Zhou Y., Tang D., Yan C., Ma J., Fang K., Gao L., Ren N. (2022). ATG7-Enhanced Impaired Autophagy Exacerbates Acute Pancreatitis by Promoting Regulated Necrosis via the miR-30b-5p/CAMKII Pathway. Cell Death Discov..

[25] Chen X., Chen X., Yan D., Zhang N., Fu W., Wu M., Ge F., Wang J., Li X., Geng M. (2024). GV-971 Prevents Severe Acute Pancreatitis by Remodeling the Microbiota-Metabolic-Immune Axis. Nat. Commun..

[26] Liu J., Yan Q., Li S., Jiao J., Hao Y., Zhang G., Zhang Q., Luo F., Zhang Y., Lv Q. (2024). Integrative Metagenomic and Metabolomic Analyses Reveal the Potential of Gut Microbiota to Exacerbate Acute Pancreatitis. npj Biofilms Microbiomes.

[27] Chen X., Shi C., He M., Xiong S., Xia X. (2023). Endoplasmic Reticulum Stress: Molecular Mechanism and Therapeutic Targets. Signal Transduct. Target. Ther..

[28] Shamoon M., Deng Y., Chen Y.Q., Bhatia M., Sun J. (2016). Therapeutic Implications of Innate Immune System in Acute Pancreatitis. Expert Opin. Ther. Targets.

[29] Watanabe T., Kudo M., Strober W. (2017). Immunopathogenesis of Pancreatitis. Mucosal Immunol..

[30] Jakkampudi A., Jangala R., Reddy B.R., Mitnala S., Reddy D.N., Talukdar R. (2016). NF-κB in Acute Pancreatitis: Mechanisms and Therapeutic Potential. Pancreatology.

[31] Ali B.M., Al-Mokaddem A.K., Selim H.M.R.M., Alherz F.A., Saleh A., Hamdan A.M.E., Ousman M.S., El-Emam S.Z. (2024). Pinocembrin’s Protective Effect against Acute Pancreatitis in a Rat Model: The Correlation between TLR4/NF-κB/NLRP3 and miR-34a-5p/SIRT1/Nrf2/HO-1 Pathways. Biomed. Pharmacother..

[32] Neuhöfer P., Liang S., Einwächter H., Schwerdtfeger C., Wartmann T., Treiber M., Zhang H., Schulz H., Dlubatz K., Lesina M. (2013). Deletion of IκBα Activates RelA to Reduce Acute Pancreatitis in Mice Through Up-Regulation of Spi2A. Gastroenterology.

[33] Jiang Y., Wu H., Peng Y., He P., Qian S., Lin H., Chen H., Qian R., Wang D., Chu M. (2024). Gastrodin Ameliorates Acute Pancreatitis by Modulating Macrophage Inflammation Cascade via Inhibition the P38/NF-κB Pathway. Int. Immunopharmacol..

[34] Mahapatra S.J., Garg P.K. (2025). Organ Failure and Prediction of Severity in Acute Pancreatitis. Gastroenterol. Clin. N. Am..

[35] Mofidi R., Duff M.D., Wigmore S.J., Madhavan K.K., Garden O.J., Parks R.W. (2006). Association between Early Systemic Inflammatory Response, Severity of Multiorgan Dysfunction and Death in Acute Pancreatitis. Br. J. Surg..

[36] Zhang R., Zhu S., Shi L., Zhang H., Xu X., Xiang B., Wang M. (2025). Automated Machine Learning for Early Prediction of Systemic Inflammatory Response Syndrome in Acute Pancreatitis. BMC Med. Inform. Decis. Mak..

[37] Bhatia M. (2009). Acute Pancreatitis as a Model of SIRS. Front. Biosci..

[38] Machicado J.D., Gougol A., Tan X., Gao X., Paragomi P., Pothoulakis I., Talukdar R., Kochhar R., Goenka M.K., Gulla A. (2021). Mortality in Acute Pancreatitis with Persistent Organ Failure Is Determined by the Number, Type, and Sequence of Organ Systems Affected. United Eur. Gastroenterol. J..

[39] Ding L., Jian L., Xu J., He Q., Wang Y., Sun C., Wang W., Sun X. (2025). Pharmacological Interventions for Acute Pancreatitis in Adults: An Overview of Systematic Reviews. J. Evid. Based Med..

[40] Marik P.E. (2009). What Is the Best Way to Feed Patients with Pancreatitis?. Curr. Opin. Crit. Care.

[41] Carnevale S., Vitale A., Razzi M., Onori C., Cornacchia G., Grispo O., Corsinovi E., Rossl L., Spinetti E., Tosi M. (2024). Non-Evidence-Based Dietary Restrictions in Hospital Nutrition and Their Impact on Malnutrition: A Narrative Review of International and National Guidelines. Dietetics.

[42] Liu H., Wang S., Wang J., Guo X., Song Y., Fu K., Gao Z., Liu D., He W., Yang L.-L. (2025). Energy Metabolism in Health and Diseases. Signal Transduct. Target. Ther..

[43] Li X.-Y., He C., Zhu Y., Lu N.-H. (2020). Role of Gut Microbiota on Intestinal Barrier Function in Acute Pancreatitis. World J. Gastroenterol..

[44] Ge P., Luo Y., Okoye C.S., Chen H., Liu J., Zhang G., Xu C., Chen H. (2020). Intestinal Barrier Damage, Systemic Inflammatory Response Syndrome, and Acute Lung Injury: A Troublesome Trio for Acute Pancreatitis. Biomed. Pharmacother..

[45] Liu W., Wu D.H., Wang T., Wang M., Xu Y., Ren Y., Lyu Y., Wu R. (2025). CIRP Contributes to Multiple Organ Damage in Acute Pancreatitis by Increasing Endothelial Permeability. Commun. Biol..

[46] Yazici C., Priyadarshini M., Boulay B., Dai Y., Layden B.T. (2024). Alterations in Microbiome Associated with Acute Pancreatitis. Curr. Opin. Gastroenterol..

[47] Wong W. (2017). Shaping the Gut Microbiome from the Pancreas. Sci. Signal..

[48] Fu Y., Mei Q., Yin N., Huang Z., Li B., Luo S., Xu B., Fan J., Huang C., Zeng Y. (2022). Paneth Cells Protect against Acute Pancreatitis via Modulating Gut Microbiota Dysbiosis. mSystems.

[49] Du B., Yan R., Hu X., Lou J., Zhu Y., Shao Y., Jiang H., Hao Y., Lv L. (2025). Role of Bifidobacterium Animalis Subsp. Lactis BB-12 in Mice with Acute Pancreatitis. AMB Express.

[50] Werawatganon D., Vivatvakin S., Somanawat K., Tumwasorn S., Klaikeaw N., Siriviriyakul P., Chayanupatkul M. (2023). Effects of Probiotics on Pancreatic Inflammation and Intestinal Integrity in Mice with Acute Pancreatitis. BMC Complement. Med. Ther..

[51] Wan Y.-D., Zhu R.-X., Bian Z.-Z., Sun T.-W. (2021). Effect of Probiotics on Length of Hospitalization in Mild Acute Pancreatitis: A Randomized, Double-Blind, Placebo-Controlled Trial. World J. Gastroenterol..

[52] Bakker O.J., Van Brunschot S., Van Santvoort H.C., Besselink M.G., Bollen T.L., Boermeester M.A., Dejong C.H., Van Goor H., Bosscha K., Ali U.A. (2014). Early versus On-Demand Nasoenteric Tube Feeding in Acute Pancreatitis. N. Engl. J. Med.

[53] Sun J., Li W., Ke L., Tong Z., Ni H., Li G., Zhang L., Nie Y., Wang X., Ye X. (2013). Early Enteral Nutrition Prevents Intra-abdominal Hypertension and Reduces the Severity of Severe Acute Pancreatitis Compared with Delayed Enteral Nutrition: A Prospective Pilot Study. World J. Surg..

[54] Rajkumar N., Karthikeyan V.S., Ali S.M., Sistla S.C., Kate V. (2013). Clear Liquid Diet vs Soft Diet as the Initial Meal in Patients With Mild Acute Pancreatitis: A Randomized Interventional Trial. Nutr. Clin. Pract..

[55] Wereszczynska-Siemiatkowska U., Swidnicka-Siergiejko A., Siemiatkowski A., Dabrowski A. (2013). Early Enteral Nutrition Is Superior to Delayed Enteral Nutrition for the Prevention of Infected Necrosis and Mortality in Acute Pancreatitis. Pancreas.

[56] Wu X.-M., Ji K.-Q., Wang H.-Y., Li G.-F., Zang B., Chen W.-M. (2010). Total Enteral Nutrition in Prevention of Pancreatic Necrotic Infection in Severe Acute Pancreatitis. Pancreas.

[57] Doley R.P., Yadav T.D., Wig J.D., Kochhar R., Singh G., Bharathy K.G.S., Kudari A., Gupta R., Gupta V., Poornachandra K.S. (2009). Enteral Nutrition in Severe Acute Pancreatitis. JOP J. Pancreas.

[58] Qin H.-L., Zheng J.-J., Tong D.-N., Chen W.-X., Fan X.-B., Hang X.-M., Jiang Y.-Q. (2008). Effect of Lactobacillus Plantarum Enteral Feeding on the Gut Permeability and Septic Complications in the Patients with Acute Pancreatitis. Eur. J. Clin. Nutr..

[59] Casas M., Mora J., Fort E., Aracil C., Busquets D., Galter S., Jáuregui C.E., Ayala E., Cardona D., Gich I. (2007). Total enteral nutrition vs. total parenteral nutrition in patients with severe acute pancreatitis. Rev. Esp. Enferm. Dig..

[60] Petrov M.S., Kukosh M.V., Emelyanov N.V. (2006). A Randomized Controlled Trial of Enteral versus Parenteral Feeding in Patients with Predicted Severe Acute Pancreatitis Shows a Significant Reduction in Mortality and in Infected Pancreatic Complications with Total Enteral Nutrition. Dig. Surg..

[61] Targarona Modena J., Barreda Cevasco L., Arroyo Basto C., Orellana Vicuna A., Portanova Ramirez M. (2006). Total Enteral Nutrition as Prophylactic Therapy for Pancreatic Necrosis Infection in Severe Acute Pancreatitis. Pancreatology.

[62] Louie B.E., Noseworthy T., Hailey D., Gramlich L.M., Jacobs P., Warnock G.L. (2005). 2004 MacLean-Mueller Prize Enteral or Parenteral Nutrition for Severe Pancreatitis: A Randomized Controlled Trial and Health Technology Assessment. Can. J. Surg..

[63] Zhao G., Wang C.-Y., Wang F., Xiong J.-X. (2003). Clinical Study on Nutrition Support in Patients with Severe Acute Pancreatitis. World J. Gastroenterol..

[64] Gupta R., Patel K., Calder P.C., Yaqoob P., Primrose J.N., Johnson C.D. (2003). A Randomised Clinical Trial to Assess the Effect of Total Enteral and Total Parenteral Nutritional Support on Metabolic, Inflammatory and Oxidative Markers in Patients with Predicted Severe Acute Pancreatitis (APACHE II > or =6). Pancreatology.

[65] Abou-Assi S., Craig K., O’Keefe S.J.D. (2002). Hypocaloric Jejunal Feeding Is Better than Total Parenteral Nutrition in Acute Pancreatitis: Results of a Randomized Comparative Study. Am. J. Gastroenterol..

[66] Oláh A., Pardavi G., Belágyi T., Nagy A., Issekutz A., Mohamed G.E. (2002). Early Nasojejunal Feeding in Acute Pancreatitis Is Associated with a Lower Complication Rate. Nutrition.

[67] Sohail R., Mathew M., Patel K.K., Reddy S.A., Haider Z., Naria M., Habib A., Abdin Z.U., Razzaq Chaudhry W., Akbar A. (2023). Effects of Non-Steroidal Anti-Inflammatory Drugs (NSAIDs) and Gastroprotective NSAIDs on the Gastrointestinal Tract: A Narrative Review. Cureus.

[68] Huang Z., Ma X., Jia X., Wang R., Liu L., Zhang M., Wan X., Tang C., Huang L. (2020). Prevention of Severe Acute Pancreatitis with Cyclooxygenase-2 Inhibitors: A Randomized Controlled Clinical Trial. Am. J. Gastroenterol..

[69] Montaño Loza A., García Correa J., González Ojeda A., Fuentes Orozco C., Dávalos Cobián C., Rodríguez Lomelí X. (2006). Prevention of hyperamilasemia and pancreatitis after endoscopic retrograde cholangiopancreatography with rectal administration of indomethacin. Rev. Gastroenterol. Mex..

[70] Sotoudehmanesh R., Khatibian M., Kolahdoozan S., Ainechi S., Malboosbaf R., Nouraie M. (2007). Indomethacin May Reduce the Incidence and Severity of Acute Pancreatitis after ERCP. Am. J. Gastroenterol..

[71] Elmunzer B.J., Scheiman J.M., Lehman G.A., Chak A., Mosler P., Higgins P.D.R., Hayward R.A., Romagnuolo J., Elta G.H., Sherman S. (2012). A Randomized Trial of Rectal Indomethacin to Prevent Post-ERCP Pancreatitis. N. Engl. J. Med..

[72] Murray B., Carter R., Imrie C., Evans S., O’Suilleabhain C. (2003). Diclofenac Reduces the Incidence of Acute Pancreatitis after Endoscopic Retrograde Cholangiopancreatography. Gastroenterology.

[73] Otsuka T., Kawazoe S., Nakashita S., Kamachi S., Oeda S., Sumida C., Akiyama T., Ario K., Fujimoto M., Tabuchi M. (2012). Low-Dose Rectal Diclofenac for Prevention of Post-Endoscopic Retrograde Cholangiopancreatography Pancreatitis: A Randomized Controlled Trial. J. Gastroenterol..

[74] Zhao X., Bao J., Hu C., Ding H., Liu X., Mei Q., Xu J. (2014). Effect of Diclofenac on the Levels of Lipoxin A4 and Resolvin D1 and E1 in the Post-ERCP Pancreatitis. Dig. Dis. Sci..

[75] Tomoda T., Kato H., Miyamoto K., Matsumi A., Ueta E., Fujii Y., Saragai Y., Yamazaki T., Uchida D., Matsumoto K. (2021). Efficacy of Low Dose Rectal Diclofenac for Preventing Post-Endoscopic Retrograde Cholangiopancreatography Pancreatitis: Propensity Score-Matched Analysis. Dig. Endosc..

[76] Werge M., Novovic S., Schmidt P.N., Gluud L.L. (2016). Infection Increases Mortality in Necrotizing Pancreatitis: A Systematic Review and Meta-Analysis. Pancreatology.

[77] De Campos T., Assef J.C., Rasslan S. (2006). Questions about the Use of Antibiotics in Acute Pancreatitis. World J. Emerg. Surg..

[78] Sun E., Tharakan M., Kapoor S., Chakravarty R., Salhab A., Buscaglia J.M., Nagula S. (2013). Poor Compliance with ACG Guidelines for Nutrition and Antibiotics in the Management of Acute Pancreatitis: A North American Survey of Gastrointestinal Specialists and Primary Care Physicians. JOP J. Pancreas.

[79] Isenmann R., Rünzi M., Kron M., Kahl S., Kraus D., Jung N., Maier L., Malfertheiner P., Goebell H., Beger H.G. (2004). Prophylactic Antibiotic Treatment in Patients with Predicted Severe Acute Pancreatitis: A Placebo-Controlled, Double-Blind Trial. Gastroenterology.

[80] Wen Y., Xu L., Zhang D., Sun W., Che Z., Zhao B., Chen Y., Yang Z., Chen E., Ni T. (2023). Effect of Early Antibiotic Treatment Strategy on Prognosis of Acute Pancreatitis. BMC Gastroenterol..

[81] Guo D., Dai W., Shen J., Zhang M., Shi Y., Jiang K., Guo L. (2022). Assessment of Prophylactic Carbapenem Antibiotics Administration for Severe Acute Pancreatitis: An Updated Systematic Review and Meta-Analysis. Digestion.

[82] Bassi C., Falconi C., Casetti L., Valerio A., Caldiron E., Butturini G., Pederzoli P. (1999). Antibiotics in Severe Pancreatitis: The Current Status. HPB.

[83] Røkke O., Bache Harbitz T., Liljedal J., Pettersen T., Fetvedt T., Øystein Heen L., Skreden K., Viste A. (2007). Early Treatment of Severe Pancreatitis with Imipenem: A Prospective Randomized Clinical Trial. Scand. J. Gastroenterol..

[84] Villatoro E., Bassi C., Larvin M. (2006). Antibiotic Therapy for Prophylaxis against Infection of Pancreatic Necrosis in Acute Pancreatitis. Cochrane Database Syst. Rev..

[85] Villatoro E., Mulla M., Larvin M. (2010). Antibiotic Therapy for Prophylaxis against Infection of Pancreatic Necrosis in Acute Pancreatitis. Cochrane Database Syst. Rev..

[86] Nordback I., Sand J., Saaristo R., Paajanen H. (2001). Early Treatment with Antibiotics Reduces the Need for Surgery in Acute Necrotizing Pancreatitis--a Single-Center Randomized Study. J. Gastrointest. Surg..

[87] Räty S., Sand J., Pulkkinen M., Matikainen M., Nordback I. (2001). Post-ERCP Pancreatitis: Reduction by Routine Antibiotics. J. Gastrointest. Surg..

[88] Saraiva M., O’Garra A. (2010). The Regulation of IL-10 Production by Immune Cells. Nat. Rev. Immunol..

[89] Berney T., Gasche Y., Robert J., Jenny A., Mensi N., Grau G., Vermeulen B., Morel P. (1999). Serum Profiles of Interleukin-6, Interleukin-8, and Interleukin-10 in Patients with Severe and Mild Acute Pancreatitis. Pancreas.

[90] Pezzilli R., Billi P., Miniero R., Barakat B. (1997). Serum Interleukin-10 in Human Acute Pancreatitis. Dig. Dis. Sci..

[91] Devière J., Le Moine O., Van Laethem J.L., Eisendrath P., Ghilain A., Severs N., Cohard M. (2001). Interleukin 10 Reduces the Incidence of Pancreatitis after Therapeutic Endoscopic Retrograde Cholangiopancreatography. Gastroenterology.

[92] Oruc N., Ozutemiz A.O., Yukselen V., Nart D., Celik H.A., Yuce G., Batur Y. (2004). Infliximab: A New Therapeutic Agent in Acute Pancreatitis?. Pancreas.

[93] Tekin S.O., Teksoz S., Terzioglu D., Arikan A.E., Ozcevik H., Uslu E. (2015). Use of Infliximab in Treatment of Acute Pancreatitis. Bratisl. Lek. J..

[94] Malleo G., Mazzon E., Genovese T., Di Paola R., Muià C., Centorrino T., Siriwardena A.K., Cuzzocrea S. (2007). Etanercept Attenuates the Development of Cerulein-Induced Acute Pancreatitis in Mice: A Comparison with TNF-Alpha Genetic Deletion. Shock.

[95] Ohwada S., Ishigami K., Yokoyama Y., Kazama T., Masaki Y., Takahashi M., Yoshii S., Yamano H., Chiba H., Nakase H. (2023). Immune-Related Colitis and Pancreatitis Treated with Infliximab. Clin. J. Gastroenterol..

[96] Triantafillidis J.K., Cheracakis P., Hereti I.A., Argyros N., Karra E. (2000). Acute Idiopathic Pancreatitis Complicating Active Crohn’s Disease: Favorable Response to Infliximab Treatment. Am. J. Gastroenterol..

[97] Randomised Treatment of Acute Pancreatitis with Infliximab: Double-Blind, Placebo-Controlled, Multi-Centre Trial (RAPID-I). https://www.centerwatch.com.

[98] Ashraf M.A., Nookala V. (2025). Biochemistry of Platelet Activating Factor. StatPearls.

[99] Chen C., Xia S.-H., Chen H., Li X.-H. (2008). Therapy for Acute Pancreatitis with Platelet-Activating Factor Receptor Antagonists. World J. Gastroenterol..

[100] Konturek S.J., Dembinski A., Konturek P.J., Warzecha Z., Jaworek J., Gustaw P., Tomaszewska R., Stachura J. (1992). Role of Platelet Activating Factor in Pathogenesis of Acute Pancreatitis in Rats. Gut.

[101] Emanuelli G., Montrucchio G., Dughera L., Gaia E., Lupia E., Battaglia E., De Martino A., De Giuli P., Gubetta L., Camussi G. (1994). Role of Platelet Activating Factor in Acute Pancreatitis Induced by Lipopolysaccharides in Rabbits. Eur. J. Pharmacol..

[102] Lane J.S., Todd K.E., Gloor B., Chandler C.F., Kau A.W., Ashley S.W., Reber H.A., McFadden D.W. (2001). Platelet Activating Factor Antagonism Reduces the Systemic Inflammatory Response in a Murine Model of Acute Pancreatitis. J. Surg. Res..

[103] Johnson C.D. (1999). Platelet-Activating Factor and Platelet-Activating Factor Antagonists in Acute Pancreatitis. Dig. Surg..

[104] Kingsnorth A.N., Galloway S.W., Formela L.J. (1995). Randomized, Double-Blind Phase II Trial of Lexipafant, a Platelet-Activating Factor Antagonist, in Human Acute Pancreatitis. J. Br. Surg..

[105] McKay C.J., Curran F., Sharples C., Baxter J.N., Imrie C.W. (1997). Prospective Placebo-Controlled Randomized Trial of Lexipafant in Predicted Severe Acute Pancreatitis. Br. J. Surg..

[106] Johnson C.D. (2001). Double Blind, Randomised, Placebo Controlled Study of a Platelet Activating Factor Antagonist, Lexipafant, in the Treatment and Prevention of Organ Failure in Predicted Severe Acute. Gut.

[107] Raggio M.J., Morris P.E. (2004). Drotrecogin Alfa. Drugs Today.

[108] Lindstrom O., Kylanpaa L., Mentula P., Puolakkainen P., Kemppainen E., Haapiainen R., Fernandez J.A., Griffin J.H., Repo H., Petaja J. (2006). Upregulated but Insufficient Generation of Activated Protein C Is Associated with Development of Multiorgan Failure in Severe Acute Pancreatitis. Crit. Care.

[109] Jamdar S., Babu B.I., Nirmalan M., Jeziorska M., McMahon R.F., Siriwardena K. (2008). Activated Protein c in L-Arginine-Induced Experimental Acute Pancreatitis. Pancreas.

[110] Alsfasser G. (2006). Decreased Inflammation and Improved Survival with Recombinant Human Activated Protein C Treatment in Experimental Acute Pancreatitis. Arch. Surg..

[111] Yamanel L., Mas M.R., Comert B., Isik A.T., Aydin S., Mas N., Deveci S., Ozyurt M., Tasci I., Unal T. (2005). The Effect of Activated Protein C on Experimental Acute Necrotizing Pancreatitis. Crit. Care.

[112] Machała W., Wachowicz N., Komorowska A., Gaszyński W. (2004). The Use of Drotrecogin Alfa (Activated) in Severe Sepsis during Acute Pancreatitis—Two Case Studies. Med. Sci. Monit..

[113] Lanzillotta M., Vujasinovic M., Löhr J.-M., Della Torre E. (2025). Update on Autoimmune Pancreatitis and IgG4-Related Disease. United Eur. Gastroenterol. J..

[114] Miranda C.J., Mason J.M., Babu B.I., Sheen A.J., Eddleston J.M., Parker M.J., Pemberton P., Siriwardena A.K. (2015). Twenty-Four Hour Infusion of Human Recombinant Activated Protein C (Xigris) Early in Severe Acute Pancreatitis: The XIG-AP 1 Trial. Pancreatology.

[115] Bernard G.R., Vincent J.-L., Laterre P.-F., LaRosa S.P., Dhainaut J.-F., Lopez-Rodriguez A., Steingrub J.S., Garber G.E., Helterbrand J.D., Ely E.W. (2001). Efficacy and Safety of Recombinant Human Activated Protein C for Severe Sepsis. N. Engl. J. Med..

[116] Kuraishi Y., Uehara T., Watanabe T., Ashihara N., Ozawa M., Kanai K., Kawa S. (2020). Corticosteroids Prevent the Progression of Autoimmune Pancreatitis to Chronic Pancreatitis. Pancreatology.

[117] Hart P.A., Topazian M.D., Witzig T.E., Clain J.E., Gleeson F.C., Klebig R.R., Levy M.J., Pearson R.K., Petersen B.T., Smyrk T.C. (2013). Treatment of Relapsing Autoimmune Pancreatitis with Immunomodulators and Rituximab: The Mayo Clinic Experience. Gut.

[118] Masaki Y., Nakase H., Tsuji Y., Nojima M., Shimizu K., Mizuno N., Ikeura T., Uchida K., Ido A., Kodama Y. (2021). The Clinical Efficacy of Azathioprine as Maintenance Treatment for Autoimmune Pancreatitis: A Systematic Review and Meta-Analysis. J. Gastroenterol..

[119] Steinberg W., Tenner S. (1994). Acute Pancreatitis. N. Engl. J. Med..

[120] Leppäniemi A., Tolonen M., Tarasconi A., Segovia-Lohse H., Gamberini E., Kirkpatrick A.W., Ball C.G., Parry N., Sartelli M., Wolbrink D. (2019). 2019 WSES Guidelines for the Management of Severe Acute Pancreatitis. World J. Emerg. Surg..

[121] Crockett S.D., Wani S., Gardner T.B., Falck-Ytter Y., Barkun A.N., Crockett S., Falck-Ytter Y., Feuerstein J., Flamm S., Gellad Z. (2018). American Gastroenterological Association Institute Guideline on Initial Management of Acute Pancreatitis. Gastroenterology.

[122] de-Madaria E., Herrera-Marante I., González-Camacho V., Bonjoch L., Quesada-Vázquez N., Almenta-Saavedra I., Miralles-Maciá C., Acevedo-Piedra N.G., Roger-Ibáñez M., Sánchez-Marin C. (2018). Fluid Resuscitation with Lactated Ringer’s Solution vs Normal Saline in Acute Pancreatitis: A Triple-Blind, Randomized, Controlled Trial. United Eur. Gastroenterol. J..

[123] Jiang J., Wang R., Song P., Peng Q., Jin X., Li B., Ni J., Shen J., Bao J., Wu Z. (2025). Lactate Facilitates Pancreatic Repair Following Acute Pancreatitis by Promoting Reparative Macrophage Polarization. Cell. Mol. Gastroenterol. Hepatol..

[124] Hoque R., Farooq A., Ghani A., Gorelick F., Mehal W.Z. (2014). Lactate Reduces Liver and Pancreatic Injury in Toll-Like Receptor– and Inflammasome-Mediated Inflammation via GPR81-Mediated Suppression of Innate Immunity. Gastroenterology.

[125] Agarwal S., Goswami P., Poudel S., Gunjan D., Singh N., Yadav R., Kumar U., Pandey G., Saraya A. (2023). Acute Pancreatitis Is Characterized by Generalized Intestinal Barrier Dysfunction in Early Stage. Pancreatology.

[126] Chen X., Zhao H.-X., Bai C., Zhou X.-Y. (2017). Blockade of High-Mobility Group Box 1 Attenuates Intestinal Mucosal Barrier Dysfunction in Experimental Acute Pancreatitis. Sci. Rep..

[127] Mei Q., Hu J., Huang Z., Fan J., Huang C., Lu Y., Wang X., Zeng Y. (2021). Pretreatment with Chitosan Oligosaccharides Attenuate Experimental Severe Acute Pancreatitis via Inhibiting Oxidative Stress and Modulating Intestinal Homeostasis. Acta Pharmacol. Sin..

[128] Lakananurak N., Gramlich L. (2020). Nutrition Management in Acute Pancreatitis: Clinical Practice Consideration. World J. Clin. Cases.

[129] Dancu G., Tarta C., Socaciu C., Bende F., Danila M., Sirli R., Sporea I., Miutescu B., Popescu A. (2023). Unraveling the Metabolic Changes in Acute Pancreatitis: A Metabolomics-Based Approach for Etiological Differentiation and Acute Biomarker Discovery. Biomolecules.

[130] Mahajan U.M., Weiss F.U., Lerch M.M., Mayerle J., Beger H.G., Büchler M.W., Hruban R.H., Mayerle J., Neoptolemos J.P., Shimosegawa T., Warshaw A.L., Whitcomb D.C., Zhao Y., Groß C. (2023). Molecular, Biochemical, and Metabolic Abnormalities of Acute Pancreatitis. The Pancreas.

[131] Wasyluk W., Zwolak A. (2021). Metabolic Alterations in Sepsis. J. Clin. Med..

[132] Gianotti L., Meier R., Lobo D.N., Bassi C., Dejong C.H.C., Ockenga J., Irtun O., MacFie J. (2009). ESPEN Guidelines on Parenteral Nutrition: Pancreas. Clin. Nutr..

[133] Meyers C., Rigassio Radler D., Zelig R.S. (2023). Impact of Solid Food Provision within 24 Hours of Hospital Admission on Clinical Outcomes for Adult Patients with Acute Pancreatitis: A Literature Review. Nutr. Clin. Pract..

[134] Arvanitakis M., Ockenga J., Bezmarevic M., Gianotti L., Krznarić Ž., Lobo D.N., Löser C., Madl C., Meier R., Phillips M. (2020). ESPEN Guideline on Clinical Nutrition in Acute and Chronic Pancreatitis. Clin. Nutr..

[135] Windsor A.C., Kanwar S., Li A.G., Barnes E., Guthrie J.A., Spark J.I., Welsh F., Guillou P.J., Reynolds J.V. (1998). Compared with Parenteral Nutrition, Enteral Feeding Attenuates the Acute Phase Response and Improves Disease Severity in Acute Pancreatitis. Gut.

[136] Feng P., He C., Liao G., Chen Y. (2017). Early Enteral Nutrition versus Delayed Enteral Nutrition in Acute Pancreatitis: A PRISMA-Compliant Systematic Review and Meta-Analysis. Medicine.

[137] Ramanathan M., Aadam A.A. (2019). Nutrition Management in Acute Pancreatitis. Nutr. Clin. Pract..

[138] Gezer A., Üstündağ H., Özkaraca M., Sari E.K., Gür C. (2024). Therapeutic Effects of Resveratrol and β-Carotene on L-Arginine-Induced Acute Pancreatitis through Oxidative Stress and Inflammatory Pathways in Rats. Sci. Rep..

[139] Kim D.-U., Kweon B., Oh J.-Y., Noh G.-R., Lim Y., Yu J., Kim M.-J., Kim D.-G., Park S.-J., Bae G.-S. (2025). Curcumin Ameliorates Cerulein-induced Chronic Pancreatitis through Nrf-2/HO-1 Signaling. Mol. Med. Rep..

[140] Zhang X., Yang G., Chen Y., Mu Z., Zhou H., Zhang L. (2022). Resveratrol Pre-Treatment Alleviated Caerulein-Induced Acute Pancreatitis in High-Fat Diet-Feeding Mice via Suppressing the NF-κB Proinflammatory Signaling and Improving the Gut Microbiota. BMC Complement. Med. Ther..

[141] Wang Y., Bu C., Wu K., Wang R., Wang J. (2019). Curcumin Protects the Pancreas from Acute Pancreatitis via the Mitogen-activated Protein Kinase Signaling Pathway. Mol. Med. Rep..

[142] Chegini M., Sadeghi A., Zaeri F., Zamani M., Hekmatdoost A. (2023). Nano-curcumin Supplementation in Patients with Mild and Moderate Acute Pancreatitis: A Randomized, Placebo-controlled Trial. Phytother. Res..

[143] Jiang H., Liu J., Xu Z., Song Q., Tao J., Zhu H., Li Q., Li L. (2025). Quercetin Alleviates Acute Pancreatitis by Modulating Glycolysis and Mitochondrial Function via PFKFB3 Inhibition. Cell. Mol. Life Sci..

[144] Jiang Z., Lhamo G., Ma M., Ye X., Chen J., He Y., Xu J., Huang L. (2025). Quercetin as a Therapeutic Agent for Acute Pancreatitis: A Comprehensive Review of Antioxidant, Anti-Inflammatory, and Immunomodulatory Mechanisms. Front. Pharmacol..

[145] Wiyono T., Nisa K., Handayani S., Windarsih A., Hayati S.N., Wulanjati M.P., Sholikhah E.N., Pratiwi W.R. (2023). Ameliorative Effect of Quercetin on Pancreatic Damage in Rodent: A Meta-Analysis. Egypt. J. Basic Appl. Sci..

[146] Al-Leswas D., Eltweri A.M., Chung W.-Y., Arshad A., Stephenson J.A., Al-Taan O., Pollard C., Fisk H.L., Calder P.C., Garcea G. (2020). Intravenous Omega-3 Fatty Acids Are Associated with Better Clinical Outcome and Less Inflammation in Patients with Predicted Severe Acute Pancreatitis: A Randomised Double Blind Controlled Trial. Clin. Nutr..

[147] Dunbar R.L., Gaudet D., Davidson M., Rensfeldt M., Yang H., Nilsson C., Kvarnström M., Oscarsson J. (2020). Omega-3 Fatty Acid Exposure with a Low-Fat Diet in Patients with Past Hypertriglyceridemia-Induced Acute Pancreatitis; an Exploratory, Randomized, Open-Label Crossover Study. Lipids Health Dis..

[148] Robles L., Vaziri N.D., Ichii H. (2013). Role of Oxidative Stress in the Pathogenesis of Pancreatitis: Effect of Antioxidant Therapy. Pancreat. Disord. Ther..

[149] Xia C.-C., Chen H.-T., Deng H., Huang Y.-T., Xu G.-Q. (2024). Reactive Oxygen Species and Oxidative Stress in Acute Pancreatitis: Pathogenesis and New Therapeutic Interventions. World J. Gastroenterol..

[150] Curran F.J., Sattar N., Talwar D., Baxter J.N., Imrie C.W. (2000). Relationship of Carotenoid and Vitamins A and E with the Acute Inflammatory Response in Acute Pancreatitis. Br. J. Surg..

[151] Morris-Stiff G.J., Bowrey D.J., Oleesky D., Davies M., Clark G.W.B., Puntis M.C.A. (1999). The Antioxidant Profiles of Patients with Recurrent Acute and Chronic Pancreatitis. Am. J. Gastroenterol..

[152] Firdous S.M., Pal S., Mandal S., Sindhu R.K. (2025). Antioxidants in Inflammatory Diseases. Antioxidants.

[153] Bhol N.K., Bhanjadeo M.M., Singh A.K., Dash U.C., Ojha R.R., Majhi S., Duttaroy A.K., Jena A.B. (2024). The Interplay between Cytokines, Inflammation, and Antioxidants: Mechanistic Insights and Therapeutic Potentials of Various Antioxidants and Anti-Cytokine Compounds. Biomed. Pharmacother..

[154] Blagov A.V., Summerhill V.I., Sukhorukov V.N., Zhigmitova E.B., Postnov A.Y., Orekhov A.N. (2024). Potential Use of Antioxidants for the Treatment of Chronic Inflammatory Diseases. Front. Pharmacol..

[155] Sateesh J., Bhardwaj P., Singh N., Saraya A. (2009). Effect of Antioxidant Therapy on Hospital Stay and Complications in Patients with Early Acute Pancreatitis: A Randomised Controlled Trial. Trop. Gastroenterol..

[156] Bansal D., Bhalla A., Bhasin D., Pandhi P., Sharma N., Rana S., Malhotra S. (2011). Safety and Efficacy of Vitamin-Based Antioxidant Therapy in Patients with Severe Acute Pancreatitis: A Randomized Controlled Trial. Saudi J. Gastroenterol..

[157] Antunes D., Gonçalves S.M., Matzaraki V., Rodrigues C.S., Gonçales R.A., Rocha J., Sáiz J., Marques A., Torrado E., Silvestre R. (2023). Glutamine Metabolism Supports the Functional Activity of Immune Cells against Aspergillus Fumigatus. Microbiol. Spectr..

[158] Guo C., You Z., Shi H., Sun Y., Du X., Palacios G., Guy C., Yuan S., Chapman N.M., Lim S.A. (2023). SLC38A2 and Glutamine Signalling in cDC1s Dictate Anti-Tumour Immunity. Nature.

[159] Stehle P., Kuhn K.S. (2015). Glutamine: An Obligatory Parenteral Nutrition Substrate in Critical Care Therapy. BioMed Res. Int..

[160] Asrani V., Chang W.K., Dong Z., Hardy G., Windsor J.A., Petrov M.S. (2013). Glutamine Supplementation in Acute Pancreatitis: A Meta-Analysis of Randomized Controlled Trials. Pancreatology.

[161] Jeurnink S.M., Nijs M.M., Prins H.a.B., Greving J.P., Siersema P.D. (2015). Antioxidants as a Treatment for Acute Pancreatitis: A Meta-Analysis. Pancreatology.

[162] Xue P., Deng L.-H., Xia Q., Zhang Z.-D., Hu W.-M., Yang X.-N., Song B., Huang Z.-W. (2008). Impact of Alanyl-Glutamine Dipeptide on Severe Acute Pancreatitis in Early Stage. World J. Gastroenterol..

[163] Liu J., Huang L., Luo M., Xia X. (2019). Bacterial Translocation in Acute Pancreatitis. Crit. Rev. Microbiol..

[164] Glaubitz J., Wilden A., Frost F., Ameling S., Homuth G., Mazloum H., Rühlemann M.C., Bang C., Aghdassi A.A., Budde C. (2023). Activated Regulatory T-Cells Promote Duodenal Bacterial Translocation into Necrotic Areas in Severe Acute Pancreatitis. Gut.

[165] Zhang C., Chen S., Wang Z., Zhang J., Yu W., Wang Y., Si W., Zhang Y., Zhang Y., Liang T. (2025). Exploring the Mechanism of Intestinal Bacterial Translocation after Severe Acute Pancreatitis: The Role of Toll-like Receptor 5. Gut Microbes.

[166] Sanders M.E., Merenstein D.J., Reid G., Gibson G.R., Rastall R.A. (2019). Probiotics and Prebiotics in Intestinal Health and Disease: From Biology to the Clinic. Nat. Rev. Gastroenterol. Hepatol..

[167] Suez J., Zmora N., Segal E., Elinav E. (2019). The Pros, Cons, and Many Unknowns of Probiotics. Nat. Med..

[168] Qin W., Wang G., Xia Y., Song X., Xiong Z., Huang C., Gong C., Zeng Y., Ai L. (2024). The Role of Probiotic Foods in Acute Pancreatitis: Current Status and Future Directions. Curr. Opin. Food Sci..

[169] Zhao Y., Zhang R., Wang S., Yang C., Wang Y., Fan H., Yang M. (2024). Observation on the Therapeutic Effect of Probiotics on Early Oral Feeding in the Treatment of Severe Acute Pancreatitis. Front. Med..

[170] Oláh A., Belágyi T., Issekutz A., Gamal M.E., Bengmark S. (2002). Randomized Clinical Trial of Specific Lactobacillus and Fibre Supplement to Early Enteral Nutrition in Patients with Acute Pancreatitis. Br. J. Surg..

[171] Oláh A., Belágyi T., Pótó L., Romics L., Bengmark S. (2007). Synbiotic Control of Inflammation and Infection in Severe Acute Pancreatitis: A Prospective, Randomized, Double Blind Study. Hepatogastroenterology.

[172] Timmerman H.M., Niers L.E.M., Ridwan B.U., Koning C.J.M., Mulder L., Akkermans L.M.A., Rombouts F.M., Rijkers G.T. (2007). Design of a Multispecies Probiotic Mixture to Prevent Infectious Complications in Critically Ill Patients. Clin. Nutr..

[173] Plaudis H., Pupelis G., Zeiza K., Boka V. (2012). Early Low Volume Oral Synbiotic/Prebiotic Supplemented Enteral Stimulation of the Gut in Patients with Severe Acute Pancreatitis: A Prospective Feasibility Study. Acta Chir. Belg..

